# Comparative In Vitro and In Silico Analyses of Phytochemicals From Butea monosperma for Wound-Healing Potential in Human Cells

**DOI:** 10.7759/cureus.62078

**Published:** 2024-06-10

**Authors:** Suraneni Venkata Dhruv Sudhakar Rao, Iadalin Ryntathiang, Archana Behera, Saantosh Saravanan, Monisha Prasad, Mukesh Kumar Dharmalingam Jothinathan

**Affiliations:** 1 Centre for Global Health Research, Saveetha Medical College and Hospitals, Saveetha Institute of Medical and Technical Sciences, Chennai, IND

**Keywords:** admet analysis, protein-ligand interaction, bioactive compounds, phytochemicals, molecular docking, wound healing

## Abstract

Aim

The objective of this study is to investigate the phytochemicals present in *Butea monosperma* and assess their potential for healing wounds using a computational comparative method.

Materials and methods

The phytochemical substances derived from *B. monosperma* were examined using a phytochemical test, Fourier-transform infrared (FTIR) spectroscopy, and gas chromatography-mass spectroscopy (GCMS). The chemical structures of these substances were investigated *in silico* using computational techniques to predict their wound-healing capacity. The molecular docking tests evaluate the binding strengths of the phytochemicals to specific proteins that play a major role in wound-healing mechanisms. The pharmacokinetic features of the substances were evaluated by analyzing their ADMET (absorption, distribution, metabolism, excretion, and toxicity) profiles.

Results

The computer analysis found several phytochemicals from *B. monosperma* that bind strongly to the proteins for wound healing: compounds such as hexanoic acid, 2,7-dimethyloct-7-en-5-yn-4-yl ester, 1,3,5-pentanetriol, 3-methyl-, and 2-butyne-1,4-diol. The ADMET analysis indicated favorable pharmacokinetic properties for the majority of the identified compounds, with low predicted toxicity.

Conclusion

Based on the *in silico* analysis, the phytochemicals in *B. monosperma* possess significant potential for use in wound-healing applications. These findings required additional *in vitro* and *in vivo* studies to confirm the effectiveness and safety of these drugs for improving wound healing. This study emphasizes the potential of *B. monosperma* as a source of innovative medicinal substances for wound care.

## Introduction

Wound healing is a complicated and progressive process that is divided into four distinct stages: the initial phase, which is hemostasis followed by inflammation; the proliferation phase; and the final phase, which is remodeling [1]. All of them are important to restore the appearance and functions of the skin and the affected tissues. Imminent is the process of hemostasis that involves vasoconstriction, platelet plug formation, and coagulation to curtail blood loss [2]. The next phase is called the inflammatory phase, where inflammatory molecules are released in the affected area to attract immune cells to clear the debris and prevent the formation of infection [3]. There is new blood channel formation, re-epithelialization, and fibroblasts that produce collagen and other extracellular matrix (ECM) molecules in the proliferation phase. The last phase of the morphologic changes is the remodeling phase, where there is maturation as well as reorganization of the collagen fibers, thereby increasing the tensile strength of the healing tissue [4].

Plant-derived compounds such as* Butea monosperma*,which is more commonly known for its medicinal properties, contain several phytochemical compounds that contribute to wound healing. These compounds include flavonoids, tannins, and triterpenoids, which exhibit anti-inflammatory, antimicrobial, and antioxidant activities. These properties promote tissue regeneration, reduce infection risk, and enhance overall healing processes [5-7]. For instance, aloe vera contains mannose-6-phosphate, which helps stimulate fibroblast proliferation and collagen formation, hence improving the rate of wound healing [8]. Similarly, an analysis of curcumin found in turmeric shows that the product can package powerful wound-healing, anti-inflammatory, and antioxidant effects that facilitate the regulation of oxidative stress and inflammation [9]. Further, *Centella asiatica* with triterpenoids increases collagen production and new blood vessel formation, which helps in wound healing [10].

The flame of the forest, or *B. monosperma*, is a plant species that has been used in traditional medicine for its bioactive compounds [11]. *B. monosperma* flower* *(BMF) parts contain larger amounts of phytochemicals, including flavonoids, tannins, and phenolics, resulting in heightened therapeutic values [12]. A comparison of the Fourier-transform infrared (FTIR) spectra of *B. monosperma* flower extract (BMFE) with pure compounds indicates the presence of the functional groups of these bioactive compounds [13]. The phytochemical constituents of the plant were also identified by gas chromatography-mass spectroscopy (GCMS), such as butein, chalcones, and sterols with anti-inflammatory, radical-scavenging, and antimicrobial activities [14]. These features make BMFE a good candidate for improving wound healing through various processes that include inflammation inhibition, infection control, and tissue repair.

Currently,* in silico* screening has become a strategic approach to drug discovery and development since it is diagnostic and inexpensive [15]. In this approach, the use of computational techniques helps in estimating the prospect of the bioactive compounds for therapy through an evaluation of their interaction with the biological targets. Molecular docking is one of the tools used in *in silico* screening that essentially predicts the interaction of phytochemicals to target proteins of the wound-healing process [16]. This technique is useful in determining the compounds with high selectivity to give those that can control major mechanisms in wound healing [17]. For instance, by use of molecular docking, the feasibility of given phytochemicals to act as matrix metalloproteinase (MMP) inhibitors involves crucial aspects of tissue remodeling and wound healing [18].

Furthermore, the protein 2GS2, implicated in wound healing, plays a significant role in tissue repair and regeneration. Its precise mechanisms involve cell migration, proliferation, and ECM remodeling, which is essential for restoring tissue integrity. Understanding its function aids in developing targeted therapies for accelerating wound closure and improving healing outcomes. In addition, computational analysis of the compound’s ADMET (absorption, distribution, metabolism, excretion, and toxicity) enlightens the pharmacokinetics of these compounds so that they can function optimally within a system as medicines [19].

Therefore, the use of phytochemicals from *B. monosperma* together with *in silico* screening methods could be a rich area to explore for new improved approaches to wound healing [20,21]. The passive integration of the indigenous knowledge system with pragmatic computer techniques has the potential to lessen the time required for the identification of bioactive molecules and thus enhance wound healing and patients’ quality of life.

This work aims to examine the phytochemicals found in *B. monosperma* and evaluate their ability to promote wound healing through a computational comparative approach.

## Materials and methods

Preparation of aqueous extract

The Indian plant of BMF was obtained from Chennai, Tamil Nadu, India. The BMF samples were verified by the Centre for Advanced Studies in Botany at the University of Madras, located in Chennai, India. The preparation of the BMF powder involved steps including washing to eliminate solid particles and dust using double-distilled water, followed by shade drying for three days. After drying, the BMF was then finely ground using a mechanical grinder and sifted through a sieve. Subsequently, the resulting biosorbent was preserved for future experimental use.

Extraction of aqueous extract

To prepare the BMFE, 25 g of dried BMF powder was dissolved in 100 mL of ionized water and mixed well. The mixture was kept overnight, followed by 20 minutes of heating at 60 °C. Once cooled to room temperature, it underwent filtration by using Whatman no. 1 filter paper, and the resulting BMFE was preserved at 4 °C for subsequent experimental analysis.

Phytochemical analysis

The aqueous extract of BMF was examined by qualitative analysis using various chemical methods [22].

FTIR

After extraction, the BMFE underwent characterization by FTIR spectroscopy (4000-400 cm^-1^), which successfully identified the specific functional groups present in the BMFE.

GCMS

A 1 mL of methanol solvent was mixed in 100 µL of aqueous BMFE. By using a vortex mixer, the solution was stirred for 20 seconds and then filtered through a 0.2-micron membrane filter. Subsequently, the clear extract was used for GCMS analysis. This methodology followed the procedure outlined by Ralte et al. [23].

*In silico* study

**Figure 1:**
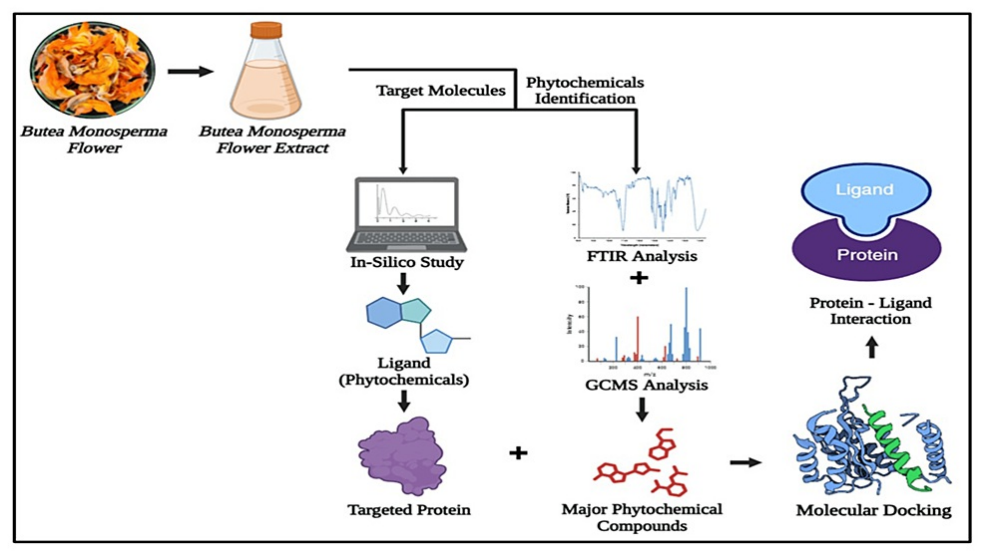
Graphical abstract of the in silico study on BMFE BMFE: *Butea monosperma* flower extract; FTIR: Fourier-transform infrared spectroscopy; GCMS: gas chromatography-mass spectroscopy

The protein structures were obtained from the Research Collaboratory for Structural Bioinformatics (RCSB) Protein Data Bank (PDB) (accessed: April 5, 2024: https://www.rcsb.org/) and validated via a build/check/repair model to ensure integrity, using AutoDock Tools for pdbqt file preparation. Ligands from PubChem were optimized in Avogadro and converted for docking. AutoDock4 conducted molecular docking with a fine-tuned grid, followed by cluster analysis to determine optimal binding poses. Binding interactions and affinities were analyzed in Biovia Discovery Studio (accessed: April 5, 2024: https://www.3ds.com/products/biovia/discovery-studio). *In silico* ADMET studies were performed using Swiss ADME (accessed: April 5, 2024: http://www.swissadme.ch/), assessing drug-likeness based on Lipinski's rule of five and interaction with biological components to predict therapeutic efficacy and safety. Figure 1 illustrates the graphical abstract of the* in silico* study on BMFE.

## Results

Preliminary phytochemical screening

On preliminary phytochemical screening of plant extracts, the following compounds, such as alkaloids, flavonoids, tannins, saponins, terpenoids, carbohydrates, proteins, and fatty acids, were obtained. No phenols or glycosides showed their presence in the extracts. The results are shown in Table 1.

**Table 1:** Phytochemical compounds from BMFE Keynotes: (+) present and (-) absent BMFE: *Butea monosperma* flower extract

Phytochemicals	Butea monosperma
Alkaloid	+
Flavonoid	+
Tannins	+
Saponin	+
Glycoside	-
Terpenoids	+
Phenol	-
Carbohydrates	+
Proteins	+
Fatty acids	+

FTIR of plant extract

To confirm the components and functional groups present in the plant extract, FTIR measurements were undertaken. The BMFE underwent FTIR analysis, disclosing peaks at various wavenumbers. The FTIR examination of BMFE identified six functional groups and associated chemical bonds. Specifically, the existence of compounds such as alcohols, aromatic compounds, alkyl aryl ethers, 1,4-disubstituted or 1,2,3,4-tetrasubstituted components or fluoro compounds, and halo or alkyl halides were confirmed.

**Figure 2:**
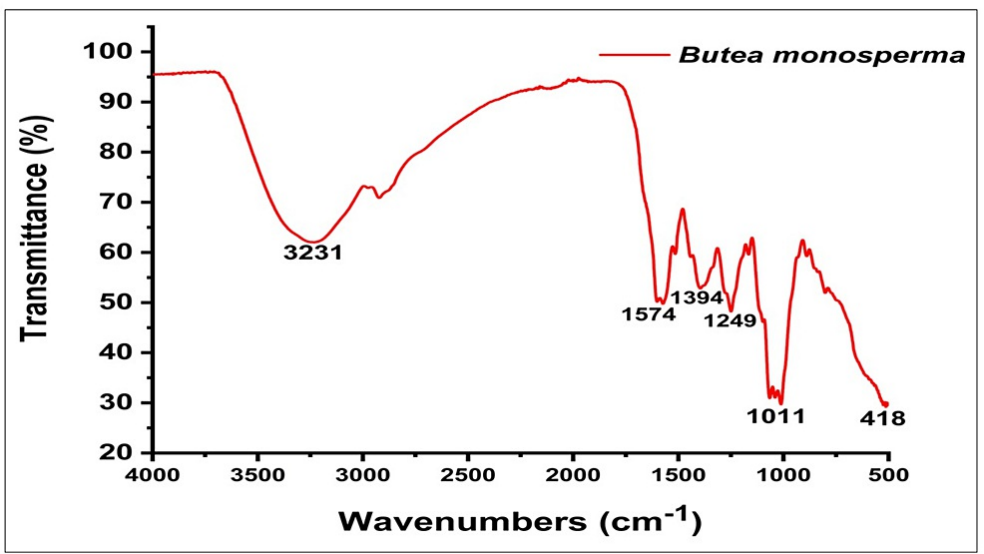
FTIR spectra of the BMFE FTIR: Fourier-transform infrared spectroscopy; BMFE: *Butea monosperma *flower extract

The FTIR evaluation of the flower extract shown in Figure 2 revealed a strong, broad peak at 3231 cm⁻¹, indicating the presence of alcohol (O-H stretching). A medium peak was noted at 1574 cm⁻¹, which proposed the presence of aromatic compounds. Another medium peak at 1394 cm⁻¹ also indicates the presence of alcohol (O-H stretch). A strong band at 1249 cm⁻¹ corresponds to the presence of alkyl aryl ether (C-O stretching). Another strong band observed at 1011 cm⁻¹ suggests the presence of either 1,4-disubstituted or 1,2,3,4-tetrasubstituted aromatic compounds (C-H bending) or fluoro compounds. Peaks observed at 418 cm⁻¹ indicate the presence of halo compounds or alkyl halides.

GCMS analysis

**Figure 3:**
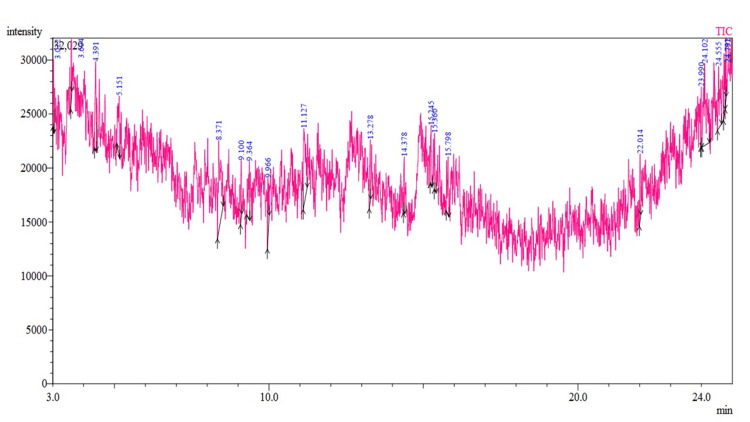
GCMS chromatogram of the flower extract of Butea monosperma GCMS: gas chromatography-mass spectroscopy

GCMS is a coherent analytical technique implemented to identify and quantify compounds present in a plant extract. Here, the samples were analyzed, and phyto-components and proteins were isolated, as illustrated in Figure 3. The overall ion current measured at every single point is displayed in this graphic. The levels of various substances in the sample are shown by the peaks. A plant compound is represented by each peak. Each peak's retention duration is identified by a label, such as 3.020, 3.384, and 5.366. The relative concentration of each plant constituent in the sample is shown by the peak's height. One of the strongest peaks, occurring at 3.020 minutes, shows a significant concentration of the related chemical. There are also noticeable peaks around the approximate time of 24.00 minutes. The area under each peak can be integrated to get the amount of every substance. Each recognized peak in the sample represents a particular compound.

Molecular docking

**Figure 4:**
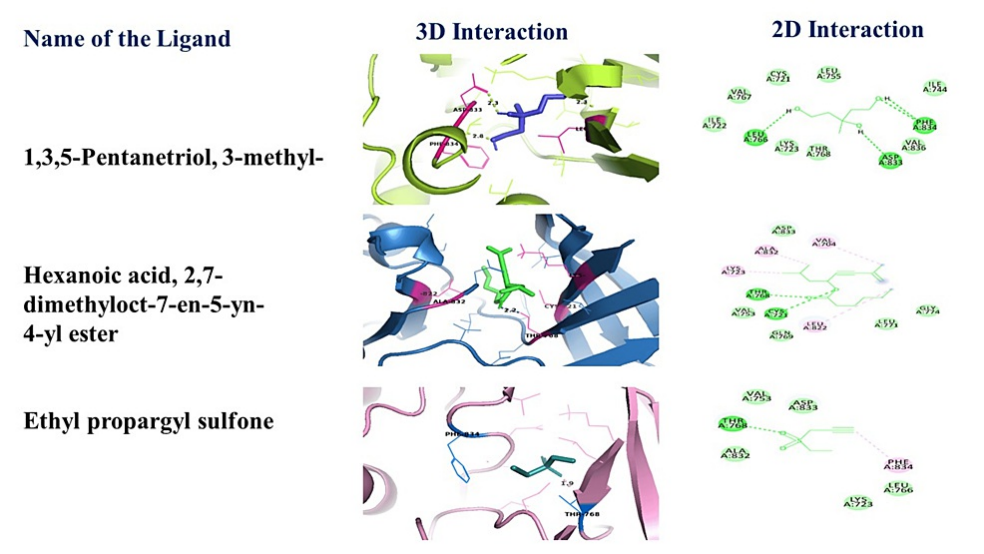
Docking (hexanoic acid with HER1) results of BMFE BMFE: *Butea monosperma *flower extract

The molecular docking approach inspects the atomic-level interactions between plant-derived phytochemicals, specifically hexanoic acid or 2,7-dimethyloct-7-en-5-yn-4-yl ester, as ligand molecules and the HER1 protein as the receptor or target molecule, as detailed in Table 2. This method is employed to investigate the binding affinity and potential of these ligands at the target protein's binding sites. In Figure 4, the molecular docking analysis of hexanoic acid with the HER1 protein displayed strong binding affinity due to the presence of hydrogen bonding, Van der Waals forces, and hydrophobic and alkyl interactions.

**Table 2:** Molecular docking on a protein with a ligand molecule

S. No.	Name of the Ligand (2GS2)	Binding Affinity Value (kcal/mol)	Distance (Å)	Hydrogen Interaction	Amino Acid Residues
1	1,3,5-Pentanetriol, 3-methyl-	-4.7	2.3 (ASP 833) 2.8 (PHE 834) 2.3 (LEU 766)	1. Van der Waals 2. Conventional hydrogen bond	1. ILE A:722, VAL A:767, CYS A:721, LEU A:755, ILE A:744, VAL A:836, THR A:768, LYS A:723 2. LEU A:766, ASP A:833, PHE A:834
2	Hexanoic acid or 2,7-dimethyloct-7-en-5-yn-4 -yl ester	-5.9	2.7 (THR 768)	1. Van der Waals 2. Conventional hydrogen bond 3. Alkyl	1. VAL A:753, GLN A:769, LEU A:771, GLY A:774, ASP A:833 2. THR A:768, CYS A:721 3. VAL A:704, ALA A:723, LEU A:822
3	Ethyl propargyl sulfone	-4.4	1.9 (THR 768)	1. Van der Waals 2. Conventional hydrogen bond 3. Alkyl	1. ALA A:832, VAL A:753, ASP A:833, LYS A:723, LEU A:766 2. THR A:768 3. PHE A:834

ADMET analysis

**Figure 5:**
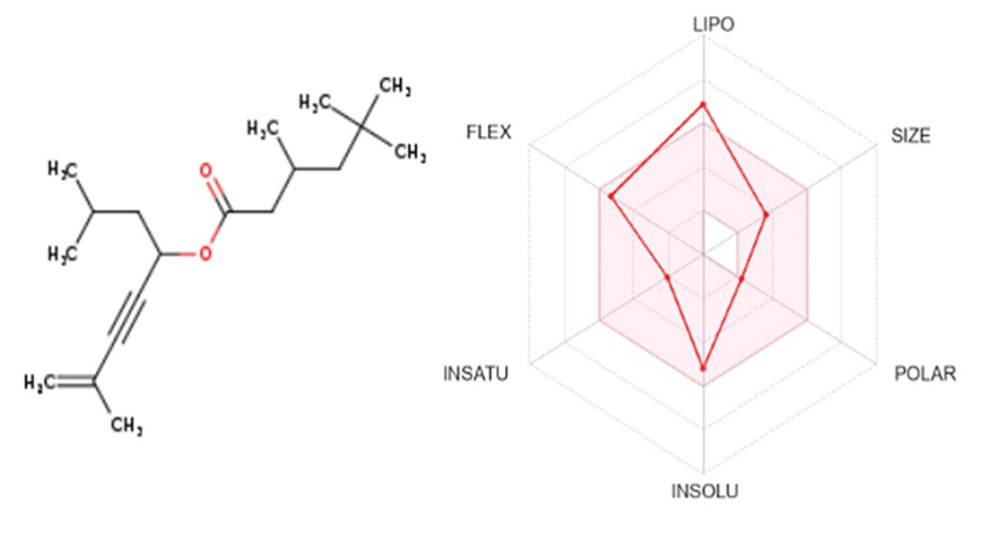
Structure of the drug

ADMET studies are designed to investigate how a chemical (2,7-dimethyloct-7-en-5-yn-4-yl ester or hexanoic acid) is processed or refined by a living organism. Based on Lipinski’s rule of five, the ADMET results for this study confirmed favorable pharmacokinetics and low potential for drug interactions (with Lipinski violation of one: MLOGP > 4.15). The molecular docking of 2,7-dimethyloct-7-en-5-yn-4-yl ester with 1,3,5-pentanetriol, 3-methyl revealed strong binding due to the presence of hydrogen bonding and hydrophobic interactions. The structure of the drug is illustrated in Figure 5. The compound's molecular weight reveals its comparatively moderate molecular size and that it is made up of 19 carbon, 32 hydrogen, and 2 oxygen atoms. The total surface area of polar atoms and the hydrogen atoms they are connected to in a molecule is described by the topological polar surface area (TPSA), which measures 26.30 Å^2^. Better cell membrane permeability and gastrointestinal bioavailability are frequently indicated by a lower TPSA. When administered orally, the chemical is well-absorbed in the gastrointestinal tract and can pass the blood-brain barrier, suggesting a possible effect on the central nervous system. The values are detailed in Table 3.

**Table 3:** ADMET properties of the drug ADMET (absorption, distribution, metabolism, excretion, and toxicity); SMILES: simplified molecular-input line-entry system; TPSA: topological polar surface area; BBB: blood-brain barrier; GI: gastrointestinal

Physiochemical Properties	
Mol wt (g/mol)	292.46 g/mol
Formula	C19H32O2
Canonical SMILES	CC(CC(OC(=O)CC(CC(C)(C)C)C)C#CC(=C)C)C
TPSA	26.30 Å^2^
BBB permeant	Yes
GI absorption	High
Lipinski violations	Yes; one violation: MLOGP>4.15
Bioavailability score	0.55
Synthetic accessibility	4.95
Water solubility	Moderately soluble

## Discussion

BMFE were separated and examined to learn more about their physiochemical characteristics, assess how much of a drug they are and how they interact with other biological substances, and consider possible therapeutic uses, particularly for the healing of wounds. The phytochemical analysis of BMFE revealed the presence of several bioactive compounds, including proteins, flavonoids, alkaloids, saponins, terpenoids, tannins, carbohydrates, fatty acids, and phenols. Additionally, GEICO sites were absent. These findings support the plants' traditional use in a range of medical applications and are in line with earlier studies, including the study conducted by Chauhan [21], where similar phytochemical components were discovered in *B. monosperma* and other medicinal plants.

The FTIR analysis provided complete clarity regarding the functional groups of the extract. Because of these functional groups, the results from various plant extracts displaying substantial medicinal value were consistent with the medium peak values at 1574 cm^-1^ and 1394 cm^-1^, indicating aromatic substances and more alcohol involvement, as well as the intense, broad peak at 3231 cm^-1^, indicative of alcohol OH stretching [24]. The important bands at 1249 and 1011 cm^-1^ confirmed the presence of C-O stretching and substituted aromatic compounds, respectively. Comparable FTIR results were found in a 2017 study by Pattanayak et al. [25] involving a range of plant species used for their antioxidant and anti-inflammatory properties. The results of the first phytochemical screening process were confirmed by GCMS analysis, which also identified and quantified the phytocomponents and proteins in the extract. This comprehensive analysis yielded a molecular fingerprint of the planned extract and found significant compounds such as hexanoic acid and 2,7-dimethyloct-7-en-5yn-4-yl ester. Research by Ambastha et al. [26] and Konappa et al. [13] has shown that these compounds are present in a variety of medicinal plants and have biological activities such as anti-inflammatory, antibacterial, and anti-cancer properties.

Molecular docking experiments were performed to understand the connection between the newly identified phytochemicals and the HER1 protein, a target associated with certain malignancies. Van der Waals forces, hydrophobic interactions, and hydrogen bonding allowed hexanoic acid to dock with the HER1 protein, which has a significant binding affinity. A previous study by Zahra et al. [27] showed that involving plant-derived substances and protein targets involved in malignancies prevents potential drug interactions, indicating that the medication is suitable for use in patients with comorbidities and for combination therapy. These encouraging results highlight the potential of hexanoic acids as a viable choice for acid-based therapeutic applications, necessitating additional preclinical and clinical research to confirm their safety and therapeutic efficacy in a range of medical settings.

ADMET experiments were performed to assess the pharmacokinetic properties of the medicines that were found. Hexanoic acid and 2,7-dimethyloct-7-en-5yn-4-yl ester were the only ones to exhibit acceptable pharmacokinetic profiles, indicating little possibility for drug interaction and advocating oral absorption. These results are comparable to those of a similar study on bioactive plant compounds: “Biological activities of *B. monosperma* (Lam) Taub. flowers on skin care cosmetics" [28]. Using molecular docking, significant binding between 1,3,5-pentanetriol, 3-methyl and 2,7-dimethyloct-7-en-5yn-4-yl ester was found. The amino acid interactions highlight crucial binding sites and affinities for hexanoic acid or 2,7-dimethyl-oct-7-en-5-yn-4-yl ester, showing enhanced binding stability. Compared to earlier studies conducted by Farooq et al. [29], these interactions provide deeper insights into molecular recognition, potentially leading to more effective therapeutic applications and drug design optimizations. This binding is thought to be caused by hydrogen bonding and hydrophobic interactions.

Limitations

The comparative i*n vitro* and *in silico* analyses of *B. monosperma* phytochemicals for wound healing has limitations. *In vitro* studies, though insightful, may not fully replicate human tissue complexity, with variability in cell lines and conditions affecting reproducibility. *In silico* analysis, while predicting molecular interactions, can oversimplify biological systems and lacks the accuracy of *in vivo* experiments. Translating these findings to clinical outcomes requires careful consideration due to differences in pharmacokinetics and bioavailability. The wound-healing process is complex, involving various cell types and pathways not fully captured in isolated studies. Thus, integrated experimental and clinical research is needed for validation.

## Conclusions

In conclusion, our *in silico*-comparative study underscores *B. monosperma's* potential for wound healing. Identifying key bioactive compounds with significant binding affinities to wound-related proteins, molecular docking, and ADMET analysis validate their efficacy and safety. This provides a foundation for future *in vitro* and *in vivo* studies to confirm therapeutic potential. Further research could involve animal models to mimic human tissue complexity and explore additional aspects of *B. monosperma's *therapeutic potential. Harnessing these phytochemicals could lead to novel wound-healing agents, offering alternatives to conventional treatments and advancing wound care through phytochemical innovation.
